# Assessing adolescents’ perceived proficiency in critically evaluating nutrition information

**DOI:** 10.1186/s12966-018-0690-4

**Published:** 2018-06-28

**Authors:** Desire Alice Naigaga, Kjell Sverre Pettersen, Sigrun Henjum, Øystein Guttersrud

**Affiliations:** 1 Department of Nursing and Health Promotion, Faculty of Health Sciences, OsloMet - Oslo Metropolitan University, Kunnskapsveien 55, 2007, KC228 Kjeller, Norway; 2 Norwegian Centre for Science Education, University of Oslo, Naturfagsenteret, Postboks 1106, Blindern, 0317 Oslo, Norway

**Keywords:** Critical nutrition literacy, Scientific literacy, Media literacy, Adolescents, Rasch analysis, Rasch modelling, Confirmatory factor analysis

## Abstract

**Background:**

Over the recent past, there has been an increase in nutrition information available to adolescents from various sources, which resulted into confusion and misinterpretation of the dietary advice. Results from international assessment frameworks such as PISA and TIMMS reflect the need for adolescents to critically appraise health information. While a number of scales measuring the critical health literacy of individuals exist; very few of these are devoted to critical nutrition literacy. More so, these scales target individuals with an advanced level of nutrition education, often gaging their proficiency in information appraisal in relation to principles of evidence-based medical research. The purpose of the present study was to examine the psychometric properties of a newly developed critical nutrition literacy scale (CNL-E) measuring adolescents’ perceived proficiency in ‘critically evaluating nutrition information from various sources’.

**Methods:**

During spring 2015, more than 1600 tenth graders aged 15–16 years from approximately 60 schools in Norway responded to the five-item questionnaire using an electronic survey system. Applying Rasch analysis approach, we examined the psychometric properties of the CNL-E scale employing the RUMM2030 statistical package. To further investigate the dimensionality of the scale and test the underlying structure, we applied multidimensional Rasch modelling using the ConQuest 4 software and confirmatory factor analysis (CFA) using the Lisrel 9.30 software.

**Results:**

In our sample, the CNL-E stood out as a valid, reliable and well-targeted scale with good overall fit to the partial credit parameterization of the polytomous unidimensional Rasch model (PCM). All the items were sufficiently statistically independent, had ordered response categories and showed acceptable individual fit to the PCM. No item displayed within-item bias or differential item functioning (DIF).

**Conclusions:**

From the observed CNL-E sum score, it is possible to draw plausible conclusions about how individuals critically evaluate nutrition information. Efforts to improve communication of nutrition information could benefit from applying validated measures such as the CNL-E scale. The CNL-E scale provides insight into how individuals without an advanced level of nutrition education, such as adolescents, determine the validity and reliability of nutrition information from various sources.

## Background

‘*For good health, increase the intake of proteins and lower the intake of fat. No, wait a second- increase the fat intake and lower the carbohydrates’*. To critically interpret such seemingly ‘contradicting’ nutrition information and dietary advice from different sources, a high level of nutrition literacy is needed.

Dietary habits acquired during early adolescence are often life-long and have a strong impact on one’s future health. Adolescents today are exposed to a vast amount of nutrition information from various sources including traditional print media such as newspapers and magazines; online media like websites, blogs, social media platforms like Facebook; advertisements on television, radios; from health experts like dietitians, doctors and from social interaction with family and peers. While increased access to nutrition information is a welcome progression in efforts to advance nutrition promotion strategies, with it has emerged an increase in the ‘confusion’ associated with having too much information, a characteristic of ‘information overload’ [1]. This points to the concept of ‘filter failure’ indicating that the strategies for deciding which information is relevant have not evolved at the same pace as the means for producing the information [2]. In addition, the assurances about the quality of information provided by these sources seems to be lagging behind [3]. These concerns have fueled the interest in exploring how individuals appraise nutrition information obtained from various sources, prior to making nutrition-related decisions.

Appraisal skills encompass the ability to interpret, filter, judge and evaluate health information obtained [4]. In the field of nutrition, these skills are associated with nutrition literacy, specifically critical nutrition literacy (CNL). Broadly, nutrition literacy refers to an individual’s capacity to access, process and understand nutrition information needed to make appropriate decisions regarding one’s nutrition [5–7]. In the domain of critical nutrition literacy, information appraisal skills are emphasized in one’s ability to evaluate the quality of the nutrition information and advice received [8]. Methodological advancement in the field of nutrition literacy has yielded a number of assessment instruments used to measure the skills and competencies associated with nutrition literacy in both clinical and non-clinical settings [9–14]. However, only a few of these measures specifically target the domain of ‘critical’ nutrition literacy [7, 15, 16]. Furthermore, these measures of critical nutrition literacy have predominantly focused on how individuals with an advanced level of education appraise information and nutrition claims based on principles of evidence-based research [16]. This focus seems to overlook how individuals without an advanced level of nutrition education, appraise and contextualize nutrition information in the media.Therefore, the aim of the present study was to examine the psychometric properties of a newly developed CNL scale measuring perceived proficiency in evaluating nutrition information from various sources, targeting individuals without an advanced level of nutrition education. Owing to the evaluation aspect of CNL, we refer to the scale as the critical nutrition literacy –evaluation (CNL-E) scale. We translated our aim into the following three hypotheses:

- H1) The CNL-E scale has acceptable overall fit to the restricted rating scale parametrization of the polytomous unidimensional Rasch model (PCM), consists of locally independent items, and represents a well targeted and reliable measurement scale.
- H2) Each item in the CNL-E scale has ordered response categories, displays no within-item bias or differential item functioning (DIF), and has acceptable individual fit to the PCM.
- H3) Using confirmatory factor analysis, the CNL-E scale has acceptable factorial validity and discriminant validity.

It follows from empirical support of the above hypotheses that reasonable claims about adolescents’ critical evaluation of nutrition information from various sources, that go beyond the observed CNL-E scale score sums, are plausible.

## Method

### Frame of reference

We randomly selected 200 schools from a list of lower secondary schools in Norway and the respective school principals were contacted by email and telephone seeking consent to volunteer in the study. From the 58 schools that accepted to participate, we collected data during the period of April to May 2015 by use of an electronic survey system from 1622 students aged 15–16 years.

### The substantive theory of the CNL-E latent variable

Basing on Nutbeam’s tripartite model of health literacy [17], nutrition literacy is categorized into three cumulative levels referred to as *functional nutrition literacy* (FNL), *interactive nutrition literacy* (INL) and *critical nutrition literacy* (CNL). FNL is concerned with basic writing and reading skills that are required to access information about nutrition. INL is comprised of the interpersonal communication and cognitive skills which enable individuals to translate and apply information in their daily lives with the aim of improving their overall nutritional status. Thirdly, CNL is concerned with higher level cognitive and social skills that enable individuals to critically appraise nutrition information and advice, as well as engage in actions that are aimed at addressing the barriers to good nutrition at individual and group levels [15, 16, 18]. The critical dimension of health literacy (CHL), which is akin to CNL, is also conceptualized as ‘judgement skills’- the ability to judge information based on factual knowledge necessary to deal with novel situations [19]. With respect to nutrition literacy, factual or declarative knowledge is characterized by an awareness of the facts and processes that pertain a certain nutrition benefit or condition [15]. Therefore, individuals that are ‘critically nutrition-literate’ are expected to meaningfuly interprete and skillfully establish how reliable, valid and credible nutrition information and dietary advice is, by comparing this information to established nutrition facts (factual knowledge).

This aspect of judging information against established factual knowledge is reflective of scientific literacy (SL), which is the capacity to apply factual scientific knowledge to identify scientific issues, explain scientific phenomena and to draw evidence-based conclusions in order to inform decisions in personal, social and global contexts [20]. Scientific knowledge in different contexts for example in the field of nutrition, provides the criteria against which information is judged. Additionally, scientific literacy is concerned with the skills that enable individuals to assess the trustworthiness (validity) of information and their willingness to participate in science-related issues, with the ideas of science as constructive, concerned and reflective citizens [20]. Therefore, it thus follows that critical nutrition literacy (CNL) is part of scientific literacy (SL) as CNL involves the application of nutrition knowledge to explain, evaluate and interpret nutrition information basing on scientific factual knowledge about nutrition and involves emancipatory action to address barriers to good nutrition [21].

Owing to the vast amount of nutrition information that is available from various sources, it is important that individuals are ‘media literate’. Media literacy encompasses the competencies and skills that enable persons to access, analyze, evaluate and produce communication in a variety of forms [22]. A proposed theory of media literacy suggests that in order for individuals to become more media literate, they must possess the capacity to comprehend information, that is to find meaning in information hosted by the media (meaning matching) and the capacity to transform information from the media and create meaning for oneself (meaning construction) [23, 24]. Central to both these inter-twined capacities of media literacy are ‘evaluation or appraisal skills’, which are suggested as one of the most relevant critical thinking skills required for the effective appraisal of messages in the media [23]. The process of critical thinking involves skillfully analyzing and synthesizing information as a guide to action and is a core component of health literacy. This is especially important if individuals are to create meaningful links between health information obtained from numerous sources in the media [25].

From the above, it is evident that evaluation skills are a crucial link between CNL, ML and SL as they enable persons to adequately identify nutrition claims, assess the consistency of nutrition information in the media and establish the validity of the underlying messages through comparison with established scientific knowledge, thereby informing their action towards overcoming barriers to good nutrition.

### The CNL-E items

The five-item CNL-E scale shown in Table 1, uses a six-point response scale anchored with the phrase ‘on a scale from ‘very difficult’ to ‘very easy’, how easy or difficult would you say it is to (1 = Very difficult, 6 = Very easy)’ The phrase is adapted from the European Health Literacy Survey Questionnaire (HLS-EU-Q47) [4]. The items were generated basing on competencies related to the process of understanding and appraising health-related information, as reflected in the integrated model of health literacy; and the category of ‘scientific enquiry’, according to the PISA framework for assessing scientific literacy [4, 26]. The sources of information were categorized into ‘traditional’ sources covering television, print sources such as newspapers, magazines and ‘online’ sources such as websites. Items 1–3 assessed the extent to which respondents felt that they could trust the nutrition information from different sources. These items explored how competent the respondents were in comprehending and interpreting nutrition information in order to maintain adequate nutritional status and prevent malnutrition. Items 4 and 5 assessed the proficiency with which respondents felt they could establish the falsifiability of nutrition claims by judging the information against basic knowledge about nutrition (facts). As the 10th grade marks the end of compulsory education in Norway, students in the 12th grade have acquired basic knowledge about nutrition (factual) and are expected to ably apply these facts while making decisions about nutrition [27].

**Table 1:** Wording of the CNL-E scale items (originally stated in Norwegian)

Item	Item wording
1	evaluate whether nutritional advice in the media (newspapers, magazines, television) is reliable?
2	consider how reliable warnings about poor nutrition are, as warnings against malnutrition?
3	consider whether information on websites for nutritional information is reliable?
4	consider what it takes a scientific nutritional claim to be valid?
5	evaluate nutritional advice in the media (newspapers, magazines, television) in a scientific way?

Note: The six-point response scale was anchored with the phrase ‘on a scale from ‘very difficult’ to ‘very easy’, how easy or difficult would you say it is to (1 = Very difficult, 6 = Very easy)’

### Person factors and data properties

In addition to the CNL-E scale, students reported on the following person factors; gender as either male or female, language predominantly spoken at home as Norwegian, Danish/Swedish (Scandinavian languages) or ‘other’, and their mother’s, father’s and own place of birth as Norway, Denmark/Sweden or ‘other’. A dummy variable, ‘cultural background’ was created with the person factor levels ‘*majority*’ if at least either the student or one of the parents were born in a Scandinavian country and ‘*minority*’ if elsewhere. The levels of linguistic and cultural background are justified by the similarities among the Scandinavian countries. Lastly, as an indicator of socioeconomic status (SES), the students reported how many books they could access at home [28]. The number of books in the home was used as an indicator of SES as research on SES and family resources shows that the aspects of the home literacy environment, such as ‘opportunity’-which includes the number of books in a home; are strongly correlated with children’s reading skills [29, 30]. Additionally, when measuring SES at student level in heterogonous groups, the number of books shows clearer differences between children from different backgrounds [31]. In order to help improve the response accuracy, a picture of how different numbers of books might appear on a bookshelf, in five groups of 10 through to 200 might look like, was included. A dichotomous variable with the levels ‘less than 100 books’ and ‘100 or more books’ was thereby defined.

#### CNL-E scale response characteristics

Of the 1622 students in the sample, 78 did not respond to any of the CNL-E items (invalid records) and 137 students (less than 10%) had one or more missing responses. Item 1 had the lowest number of missing responses (80) and item 4 had the highest number of missing responses (109). There were 75 extreme scorers in the data set; 28 of whom responded “1” to all five items and 45 of whom responded “6” to all five items. With 78 invalid records and 75 extreme scorers, there were 1469 students with valid scores available for analyses.

### Validating measurement models approach 1: Rasch analysis (RA) – Testing the empirical data up against the theoretical requirements of fundamental measurement

A measurement model describes how responses to a set of items (observed variables) reflect a unidimensional latent trait (unobserved variable), such as ‘critically evaluating nutrition information. Theoretically, Rasch models fulfill the assumptions and requirements of fundamental measurement such as unidimensionality, equal item distribution, specific objectivity and additivity [32–39]. Rasch analysis makes it possible to assess the psychometric properties of new and existing scales, by assessing whether the response patterns in the data fit the expectations of Rasch models [40]. Based on the prescriptive Rasch models, the distance between the item location (difficulty) and person location (proficiency) defines the expected probability of a certain response [41]. The polytomous unidimensional Rasch model (PCM) assumes two parameterizations, the ‘unrestricted’ partial credit parameterization (PCM) [42] or the ‘restricted’ rating scale parameterization (RSM) [43], where the latter is nested within the first. Data-model fit of ‘competing’ nested models is compared applying likelihood ratio tests (LRT) [44]. The LRT test statistic is the difference or change in deviance, which is the asymptotically chi-square (*χ*^2^) distributed statistic with degrees of freedom (*df*) equal to the difference in number of estimated parameters. A large and significant *χ*^2^ value indicates that the null hypothesis, which states that the less complex nested model or parameterization describes the data better than the more complex model or parameterization; should be rejected. If we compare models before and after discarding and adding items, we no longer have nested models and apply measures such as the Akaike Information criteria (AIC) [43–45].

#### Fit to the Rasch model

Comparing the ‘theoretically or model expected’ probabilities of responses to the ‘empirically observed’ portions, yields a formal ‘chi-square test of goodness-of-fit’. The concept ‘item discrimination’ indicates how well an item is capable of discriminating or separating between individuals with higher person location estimates along the latent trait from those with lower estimates. An ‘under-discriminating’ item is indicative of a weaker distinction between such respondents than what is expected by the RM and is indicated by a large and nonsignificant item chi-square value (*p*(*χ*^2^) < 5%) as compared to the *χ*^2^ distribution on that degrees of freedom. To account for statistical misfit that might arise owing to chance, we adjust the significance level by the number of *χ*^2^ tests applied, applying the Bonferroni adjustment [39].

#### Ordering of response categories and differential item functioning

When respondents use the rating scales as intended, ordered thresholds reflect the increasing levels of severity across each response category [40]. The different threshold locations reflect the locations at which the probability of a response in two adjacent categories is equal.Within-item bias is examined by checking for the presence of differential item functioning (DIF). DIF is indicated when individuals with the same standing on the latent trait belonging to different categories of a person factor (such as gender) have different probabilities of endorsing an item [41, 46].

#### Targeting and reliability

In a well-targeted scale, the distribution of the person estimates matches the distribution of the item threshold estimates, where either the person or item estimates are centered at 0.0 logits. Poor targeting increases the risk of extreme scores and unordered response categories. The Person Separation Index (PSI), which is analogous to Cronbach’s alpha, indicates how precise the measurement is, given unidimensionality. Values greater than 0.70 suggest better internal consistency reliability [42].

#### The assumption of local independence

The Rasch models assume locally independent items – i.e., all covariance between the items is attributed to the latent trait variable or ‘Rasch factor’. Violation of local independence is reflected as either *multidimensionality* implying that more than one latent variable influences the responses, or *response dependence* where subsets of items share further similarities than those accounted for by the latent trait variable.). Response dependence is indicated by significant correlation between item model residuals (>.30) [43, 44].

To empirically check for unidimensionality, a combined principal components analysis of residuals (PCA) and paired *t*-tests procedure is available in RUMM. If 5% or less of the *t*-tests are significant, then the proportion of instances in which two item subsets yield “significantly” different person location estimates is small enough to retain the hypothesis of unidimensionality [46–49]. Additionally, subtest structures based on theoretical or empirical assumptions of subsets of items might be formed to investigate violations of local independence [50].

Furthermore, additional tests of dimensionality can be carried out by applying multidimensional Rasch modelling in the ConQuest [51] program and confirmatory factor analysis (CFA) using Lisrel [52].

### Validating measurement models approach 2: Confirmatory factor analysis (CFA) – Covariance characteristics defining latent traits

A structural equation model involves *measurement models* that define latent variables and a *structural model* to indicate how the latent variables are related [53].

#### Model specification

**Fig. 1:**
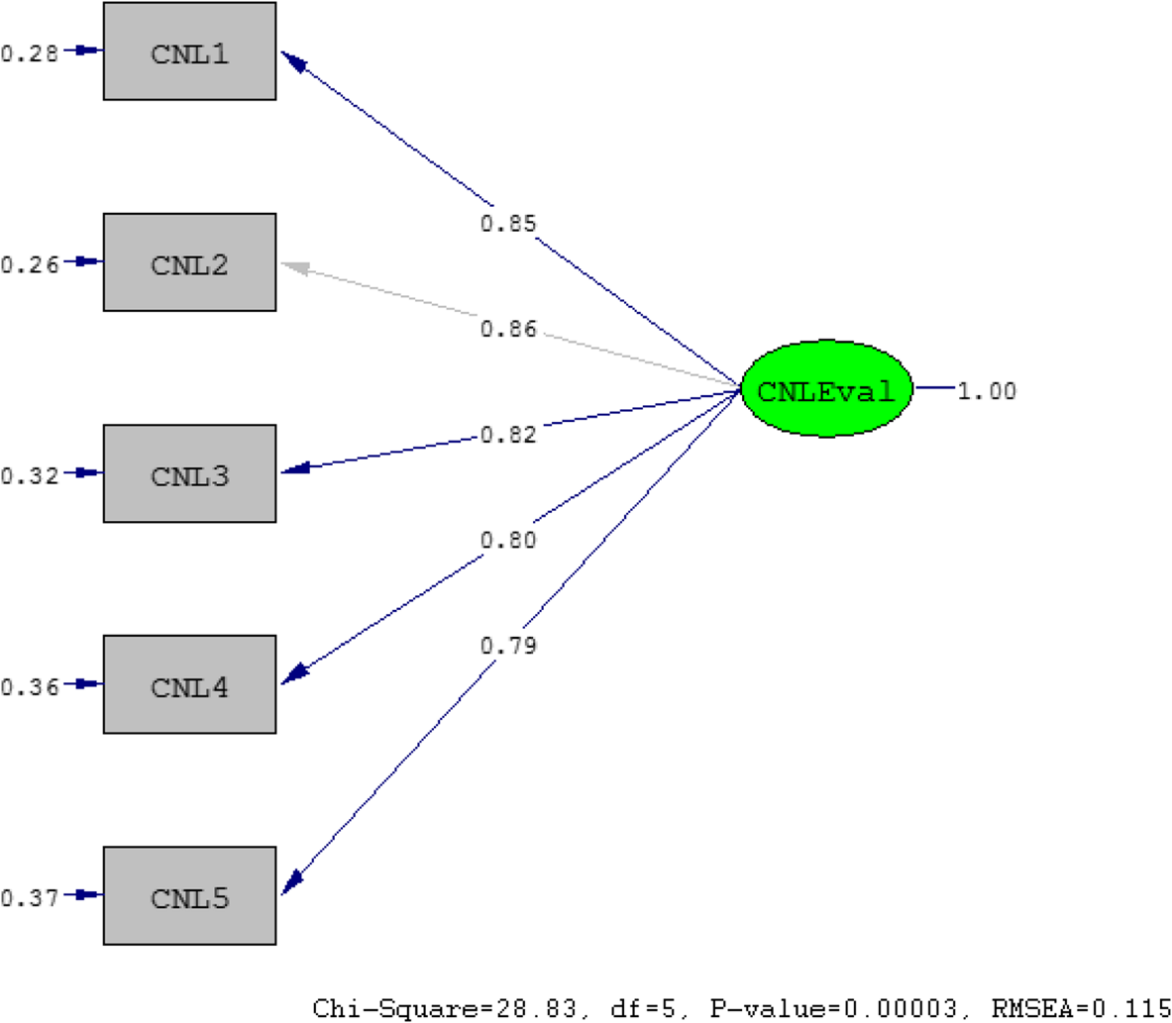
The highest factor loading (CNL2) was fixed to 1.0 (completely standardized solution). The rectangular boxes represent the observed variables (CNL1-CNL5), while the oval shape represents the unobserved latent factor, CNL-E. The single arrows pointing towards the observed variables indicate the specific variance for each of the five variables

**Fig. 2:**
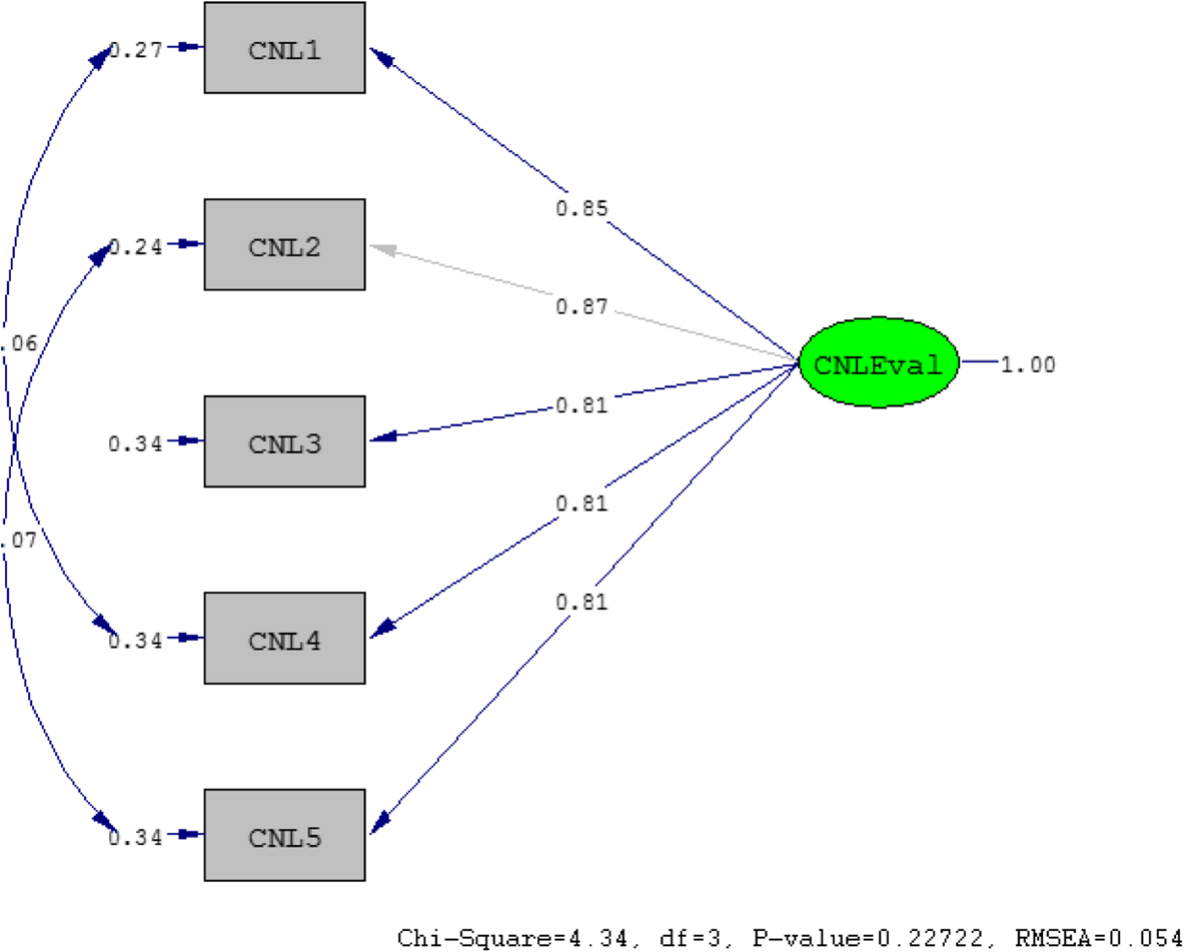
The double-headed arrows represent the uniqueness correlations between CNL1 & CNL4, and CNL2 & CNL5. The highest factor loading (CNL2) is fixed to 1.0 (completely standardized solution). The rectangular boxes represent the observed variables (CNL1-CNL5), while the oval shape represents the unobserved latent factor, CNL-E. The single arrows pointing towards the observed measured variables indicate the uniqueness, a composite of specific variance and measurement error specific to each of the five variables

A confirmatory factor model is based on theoretical assumptions, that the observed variables represent the latent variable accurately, albeit with a unique variance (error). The model in Fig. 1 demonstrates that the latent variable “critical nutrition literacy evaluation” (CNL-E) is measured by the observed variables CNL1, CNL2, CNL3, CNL4 and CNL5, taking into account the unique variance associated with each of the observed variables CNL1-CNL5. Formally, the model in Fig. 1 is a hypothesized a priori 1-factor confirmatory factor model (M1) testing the hypothesized relationship between the latent variable and the observed variables i.e.; whether the responses to the questionnaire items measure the latent variable. The factor structure for the hypothesized a priori 1-factor confirmatory factor in Fig. 2 for a post hoc modified model (M2) is conceptually diagrammed, based on modification indices suggested by Lisrel between items CNL1 and CNL4 and CNL2 and CNL5. In both figures, the variance of the latent variable (CNL-E) is fixed to 1.0 (completely standardized solutions).

#### Model identification

Refers to deciding on whether a single value for each unknown model parameter, referred to as a free parameter (FP) is obtainable from the observed data. Because latent variables are unobservable, they have no scale of their own; therefore their origin and unit of measurement can be assumed by defining the unit of the latent variable in relation to reference variable-an observed variable whose coefficient (factor loading) is fixed; and or by fixing the variance of the latent variable to 1.0, thereby assuming that it is a standardized variable.

As the observed variance-covariance matrix ***(S)*** is an “unstructured” and symmetric matrix, it contains *k*(*k* + 1)/2 unique or distinct values (DV). The single factor CFA measurement model in Fig. 1 has *k* = 5 items so there are 15 DV in ***S***. The number of free parameters (FP) to be estimated for the model in Fig. 1 were counted as following (see Table 5): 4 factor loadings (with 1 other factor loading (CNL2) fixed to 1), 5 item unique variances, 0 item unique covariances^1^ (or correlations in standardized solution) and 1 latent variable variance – a total of 10 free parameters. For a model to be identified, FP must be less than or equal to DV. Therefore, the model in Fig. 1 is “over-identified” with degrees of freedom *df* = DV-FP = 5. This means it is possible to obtain a single value for each unknown FP from the observed data and still have degrees of freedom available for estimating data-model fit.

### Model estimation

Refers to the estimation of data-model fit. Using diagonally weighted least squares (DWLS) and or maximum likelihood (ML) estimation, the FP are estimated with the aim of obtaining a model-based (implied) variance-covariance matrix (***Σ)*** with elements as close as possible to the elements in ***S*** [53, 54]. The objective of the estimation is therefore to achieve a residual variance-covariance matrix ***S*-*Σ*** where the elements are as small as possible. Because ML assumes multivariate normality, in cases of non-continous distribution, such as with observations made on ordinal variables like those obtained from rating scales as in the current study; asymptomatic distribution-free (ADF) estimators are used [55]. These include the diagonally weighted least squares (DWLS), which make no assumptions about the distribution of the observed variables. DWLS minimizes the chance over-estimating chi-square fit values and underestimating standard errors [53].

#### Model evaluation

Refers to evaluating the discrepancy between ***S*** and ***Σ***. Absolute fit indices such as the chi-square (*χ*^2^) and standardized root-mean-square residual (SRMR), are used to achieve this. A statistically significant chi-square value implies imperfect model fit and points to rejection of the model [55], therefore it is advisable to report other fit indices as they provide different information about model fit, providing more conservative and reliable evaluation of the fit to the model. The “parsimony correction indices”, such as the root mean square error of approximation (RMSEA) and “close” fit (Cfit), evaluate the discrepancy between ***S*** and ***Σ*** while penalizing complex models with many parameters [53–55]. Using ML or DWLS estimation along with the asymptotic covariance matrix, LISREL implements the mean-adjusted Santorra-Bentler scaled *χ*^2^ to adjust for non-normality. Incremental fit indices, such as the comparative fit index (CFI) and the non-normed fit index (NNFI) or Tucker-Lewis index (TLI), assess absolute or parsimonious fit relative to a baseline model hypothesizing no relationships among the variables. The latter indices are therefore rather liberal, with values greater than 0.95 for the CFI, NNFI (TLI), indicative of an acceptable model-data fit [54]. Values <.05, .05- < .08, .08–.10 imply good fit, reasonable fit (.05- < .08) and mediocre fit (.08–.10), while values >.10 indicate poor fit. An associated fit index is the C-fit value, which is a test of the closeness of fit when RMSEA < 0.05. Values greater than 0.05 indicate a good model fit [54–56].

Lastly, the critical sample (CN) statistic; which shows the size that a sample should reach in order to accept the fit of a given model on a statistical basis. Values > 200 indicate that the model is an accurate representation of the data [57].

#### Model modification

Refers to adding or removing items and/ or paths to obtain better data-model fit – that there are alternative models predicting the observed variables better. Modification indices > 3.84 indicate which previously fixed parameters should be set free (added) in order to improve model fit maximally [53]. However, this should only be done if the modifications fit the underlying theory. It is advisable that where possible, researchers test the resultant post-hoc model on a different sample as adjusting models after initial testing increases the chances of capitalizing on sampling error, in that ‘idiosyncratic characteristics of the sample may influence the modifications performed’ [58]. Furthermore, because model modifications generally result into better fitting models, there is a risk of having more data-driven than theory-driven models which are not generalizable across samples [59]. Therefore, it is important to justify any model modifications on empirical and/or conceptual grounds such as item content and violations of local independence [60].

## Results

### Rasch analysis

This section begins with a discussion of the dimensionality of the data, fit to the Rasch model, the individual item response dependency (local independence) and finally elaborates on the validity of the theoretically derived model using confirmatory factor analysis, for the latent variable ‘critically evaluating nutrition information from various sources’. All analyses run smoothly.

**Fig. 3:**
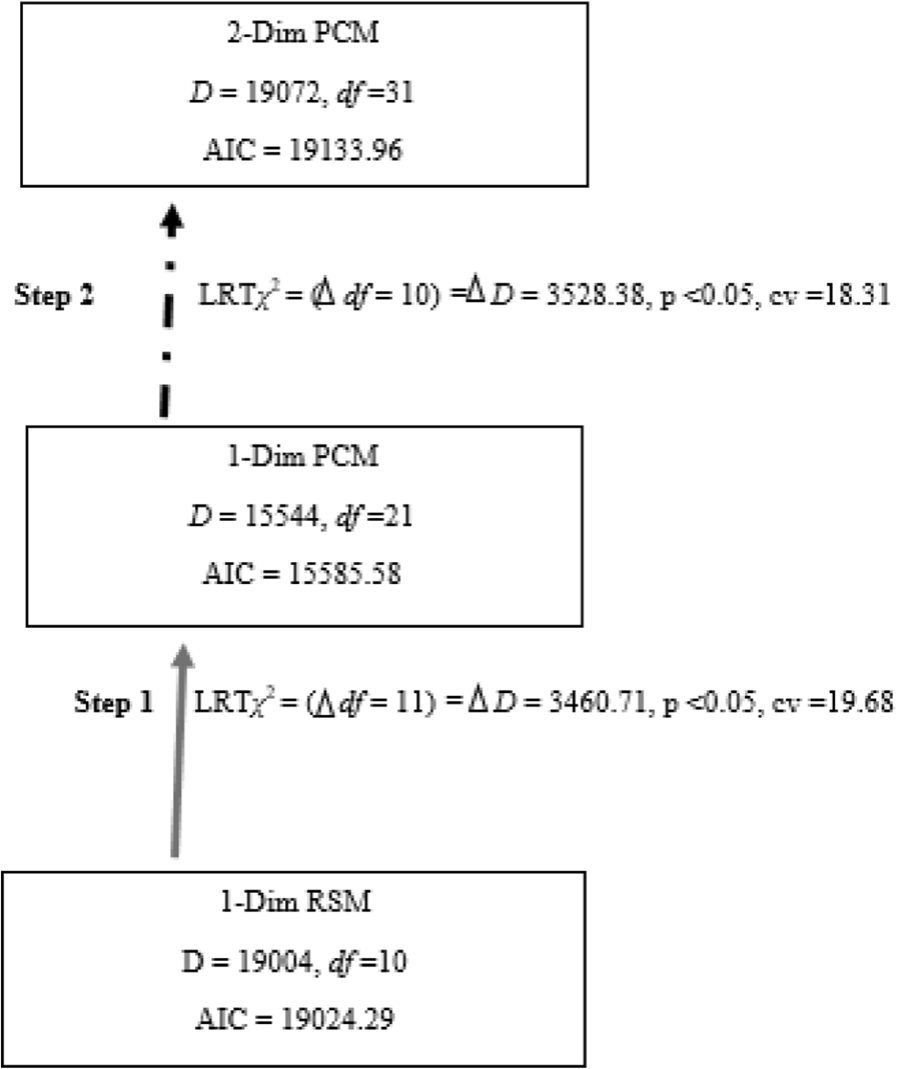
Step 1 tests the null hypothesis that the restricted rating scale parameterization (RSM) describes the data as well as the more complex partial credit parameterization (PCM) of the polytomous unidimensional Rasch model (PCM). Step 2 investigates dimensionality, comparing fit to the PCM of the 1-dimensional scale and a 2-dimensional scale. In the 2-dimensional scale, the items are categorized into two ‘sub-dimensions’ i.e., items 1, 2, 3 and items 4 and 5, based on qualitative interpretation of item content, confirmed by the PCA/*t*-test procedure in RUMM2030. The dashed arrow indicates that the 1-dim PCM is the preferred parameterization

Using ConQuest, the data was fitted to the partial credit parameterization (PCM) (deviance =15,544, number of estimated parameters = 21) and to the rating scale parameterization (RSM) (deviance = 19,004, number of estimated parameters = 10) of the unidimensional polytomous Rasch model. Comparing these nested parameterizations yielded a significant LRT chi-square statistic *χ*^2^ (Δ*df* = 11, *N* = 1469, *p* < 0.05, critical value = 19.68, implying that the PCM describe the data significantly better than the RSM. The change in deviance is asymptotically *χ*^2^ distributed (see Step 1 in Fig. 3).

Applying the principal component analysis in RUMM, the subset of items CNL4 and CNL5 loaded positively on the first principal component, while the subset of items CNL1, CNL2 and CNL3 loaded negatively on that component. These two item subsets might therefore tap into two different aspects of the overall underlying trait ‘critically evaluating nutrition information and therefore possibly define two subscales which might rank individuals differently. Regarding local independence, none of the residual correlations between any pairs of items in the scale excceded 0.3, implying that there was no significant response dependence between the items. However the presence of large negative residual correlations less than − 0.3, pointed to the presence of possible underlying dimensions (multidimensionality). Furthermore, a PCA of item residuals yielded two item sets comprised of items CNL1, CNL2 and CNL3 and items CNL4 and CNL5, respectively. Subsequently paired *t*-tests implied a sufficiently unidimensional scale as approximately 5% of the paired t-tests were significant. Using ConQuest, the data was fitted to the partial credit parametrization of the 2-dimensional polytomous Rasch model (deviance = 19,072, number of estimated parameters =31), where the two item subsets defined the two dimensions (see Step 2 in Fig. 1), and to the partial credit parametrization of the unidimensional polytomous Rasch model (deviance =15,544, number of estimated parameters = 21). Comparing these nested models yielded a non-significant LRT chi-square statistic *χ*^2^ (Δ*df* = 10, *N* = 1469) *p* < 0.01, critical value = 23.2 (Step 2 in Fig. 1) pointing to better data-model fit for the the unidimensional than the multidimensional Rasch-model.

At the individual item level, all five CNL-scale items meet the model expectations and fit well to the partial credit parametrization of the unidimensional polytomous Rasch model (PCM)as shown in Table 2 (chi-square values for N = 1469).

**Table 2:** Individual item fit statistics for the critical nutrition literacy-evaluation (CNL-E) scale (pairwise maximum likelihood estimation using RUMM)

Item	Loc	SE	Thresholds					FitRes	*χ*^2^ (*N* = 1395)	*p*(*χ*^2^)
3	− 0.171	0.04	− 3.3	− 1.8	− 0.1	1.7	3.5	−0.48	4.5	0.88
4	−0.099	0.04	−3.1	−2.0	−0.2	1.9	3.4	1.40	6.1	0.73
2	−0.076	0.04	−3.7	−2.0	−0.0	2.0	3.7	−2.14	12.4	0.19
5	0.097	0.04	−2.8	−1.8	−0.2	1.6	3.3	1.24	9.9	0.36
1	0.248	0.04	−2.7	−2.0	−0.3	1.7	3.2	−1.72	9.6	0.39

*Note:* Items sorted by location order, applying the partial credit parameterization (PCM) of the polytomous unidimensional Rasch model (PCM) was applied, *df* = 9

Estimates shown are from unidimensional Rasch analysis using RUMM2030 software showing item location point estimate (*Loc*) with standard error (*SE*). The *p*(*χ*^2^) reports the probability of observing the *χ*^2^ value on the given degrees of freedom (*df*) where *df* = *G*-1 and *G* is the number of proficiency groups applied in the formal test of fit. The *thresholds* indicate the location of the items along the latent continuum, covering the latent trait from approximately −3.7 to + 3.7 logits. The rounded average of the five thresholds are approximately − 3, − 2, − 0, 2 and 3 logits respectively

All of the items in the CNL-E scale displayed ordered response categories, implying that the CNL-E raw score produces data at the ordinal level and can be transformed to interval using Rasch-modelling.

No item displayed within-item bias (DIF) across the different person factor levels for the available person factors (age, gender, socio-economic status/books at home, linguistic background/language spoken at home and cultural background/place of birth).

With the average item location centered at 0.0 logits, the mean person location at 0.42 logits suggests a sufficiently well-targeted scale, meaning that the items in the CNL-E scale sufficiently captured the range of the latent trait within the sample. From RUMM, using the weighted maximum likelihood estimator (WMLE) high reliability indices (PSI = 0.88 for the original data set (with missing values) and Cronbach’s alpha = 0.90 for the complete data set without missing values), indicate that the CNL-E scale was a reliable measure in our sample. Likewise, estimation of the multidimensional model in ConQuest applying marginal maximum likelihood estimation (MLE), showed that both subscales in the 2-dimensional scale had MLE person separation reliability coefficients larger than 0.70 i.e.; dim1 (0.754), dim 2(70.21). Therefore, the CNL-E scale seems to measure “critical evaluation of nutrition information from various sources” sufficiently reliably in our sample.

### Confirmatory factor analysis of the underlying latent structure

Using Lisrel, we conducted a confirmatory factor analysis in which we specified the a priori one-factor (CNL-E) measurement model with five observed variables (items CNL1 – CNL5). Applying robust maximum likelihood estimation, the goodness-of-fit SRMR index was well below its target value (Table 3) and the Satorra-Bentler *χ*^2^ value was insignificant possibly owing to large sample size (*N* = 1469). The CFI and NNFI were both very high and clearly above their respective target value. As the RMSEA was above .06 the CFit was below .05. Therefore, the absolute and the incremental fit indices, as opposed to the parsimony-adjusted indices, strengthened the hypothesis of a well-fitting measurement model. The post hoc model (model M2 in Table 3), in which we added the uniqueness covariance between items CNL2 & CNL5 and between CNL1 & CNL4, as suggested by the modification indices in Lisrel; showed a significant improvement in all six fit indices as seen in Table 3. The modifications were purely data-driven and might therefore capitalize on sampling error. It is warranted that future studies define both models a priori and test whether model 2 (M2) is preferred to model 1 (M1).

**Table 3:** Model evaluation by goodness-of-fit indices (GOFI) for the a priori specified measurement model M1 in Fig. 2 and the re-specified and data-driven post hoc modified measurement model M2 in Fig. 3 (robust maximum likelihood estimation using the statistical package LISREL)

Model (M) and GOFI goodness-of-fit target value	Absolute GOFI		Parsimony-adjusted GOFI		Incremental GOFI	
	SB scaled *χ*^2^ with p-value	SRMR	RMSEA (90% CI)	CFit	CFI	NNFI
M1 (*df* = 5, *N* = 1485)	28.83, ***p*** **= .0000**	0.021	**0.115 (0.096; 0.135)**	**0.000**	0.995	0.990
M2 (*df* = 3, *N* = 1485)	4.34, *p* = .2272	0.009	0.054 (0.030; 0.081)	0.358	1.000	0.999
target value	*p* > .05	< .05	< .06 (< .05; < .08)	> .05	> .95	> .95

*Note:* M2 is the more restricted and nested model obtained from M1 by the addition of the covariance between the uniqueness variance components of items CNL1 and CNL4, and items CNL2 and CNL5

*df* = degrees of freedom, *N* = effective sample size (list wise deletion). Goodness-of-fit indices (GOFI) are classified as absolute, parsimony-adjusted and incremental: SB scaled *χ*^2^ = Satorra-Bentler scaled chi-square, SRMR = Standardized Root Mean Square Residual, RMSEA = Root Mean Square Error of Approximation, CFit = p-value for test of Close Fit (i.e., the probability that RMSEA < 0.05), CFI = Comparative Fit Index, NNFI = Non-Normed Fit Index = TLI = Tucker & Lewis fit index. Bold values imply mediocre to poor data-model fit (the SB scaled *χ*^2^ *p*-value for M1 is insignificant owing to large sample size)

Furthermore, investigation of the standardized residual matrix pointed to improved ‘local fit’ in the post hoc modified model, as indicated by a rise in the standardized residual values following the addition of parameters between the error variances as suggested by the modification indices (Table 4).

**Table 4:** Standardized residual matrices for the critical nutrition literacy evaluation (CNL-E) measurement models

Original a priori model:					
Variable	CNL1	CNL2	CNL3	CNL4	CNL5
CNL1					
CNL2	0.380				
CNL3	0.658		0.000		
CNL4	−1.668	1.627	−0.862	0.000	
CNL5	0.673	−1.380		1.390	
Modified *posthoc* model:					
Variable	CNL1	CNL2	CNL3	CNL4	CNL5
CNL1	0.000				
CNL2		0.000			
CNL3	0.959		0.000		
CNL4	0.000		−1.024	0.000	
CNL5	−0.341	0.000	0.875	0.215	0.000

*Note:* All standardized residuals of the a priori and post hoc modified models are within the accepted range of ≤ +/− 1.96. The largest values (−1.668, − 1.380, 1.390) indicate that the a priori model does not account very well for the correlations between CNL1 and CNL4, CNL2 and CNL5, and CNL4 and CNL5 respectively. Adding parameters between the error covariances of CNL1 and CNL4, and CNL2 and CNL5 in the post hoc modified model results into a decrease in the residual values, indicating better fit

Investigation of the parameter estimates of both the a-priori model (M1) and post hoc modified model are shown in Tables 5 and 6 respectively. The better fitting model-the post hoc modified model, shown in Table 6, show that all the factor loadings exceeded 0.71 and all unique variances were below 0.50, an indicator that the latent trait under study largely explained the variance in the responses to the observed variables. Taken together, the five variables measured accounted for approximately 70% of the variance in the latent factor, as indicated by a mean *R*^2^ value of 0.69. Both the a priori specified and post hoc modified models were over-identified as the difference between the number of distinct values (15) and the number of free parameters (10 and 12, respectively) were larger than 0 (*df* = 15–10 = 5 and *df* = 15–12 = 3, respectively). However, since the better fitting model- the post hoc model (M2) was tested on the same sample, there is a possibility of ‘capitalizing on sampling error’.

**Table 5:** Model identification and model estimation for the a priori measurement model in Fig. 2 (applying robust DWLS and ML using the statistical package LISREL)

Model Identification		Unstandardized solution				Completely standardized solution	
		DWLS		ML		DWLS	ML
FP	Observed variables	Estimate	(SE)	Estimate	(SE)	Estimate	Estimate
1	CNL1 factor loading	.981	(.024)	.984	(.020)	.856	.847
	CNL2 factor loading	1.000*		1.000*		.872	.861
2	CNL3 factor loading	.932	(.020)	.958	(.020)	.813	.824
3	CNL4 factor loading	.932	(.023)	.928	(.021)	.813	.799
4	CNL5 factor loading	.931	(.021)	.923	(.022)	.812	.794
5	CNL1 unique variance	.267		.282		.267	.282
6	CNL2 unique variance	.239		.259		.239	.259
7	CNL3 unique variance	.339		.320		.339	.320
8	CNL4 unique variance	.339		.362		.339	.362
9	CNL5 unique variance	.340		.369		.340	.369
	Latent variable						
12	CNL-Eval variance**	.742	(.023)	.741	(.024)	1.000	1.000

*Note*. CNL1 - CNL5 are the observed variables, CNL-Eval is the latent variable. FP = Free parameter (counting the number of free parameters to be estimated with reference to the unstandardized soultion), DWLS = Diagonally Weighted Least Squares estimation, ML = Maximum Likelihood estimation, SE = Standard Error, Factor loading = the proportion of the total variance that an item shares with the other items ie., is common to the items (a variance component accounted for by the latent variable in the model), Unique variance = the proportion of the total variance that is unique to an item (a variance component not accounted for by the latent variable model in the model i.e., the *un*modelled variance component). Additional correlation was specified between the error covariances of CNL1 and CNL4 and CNL2 and CNL5

#) Lisrel reports unique variance components as 1-*R*^2^ for both the standardized and the unstandardized solutions, where *R*^2^ is the squared standardized factor loading when the item only load on one factor

*) Factor loading constrained to 1 owing to item being used as reference or marker variable to resolve the origin and unit of measurement problem

**) The variance of the latent variable is the “covariance with itself” in the *un*standardized solution and the “correlation with itself” in the standardized solution. The latter is always 1

**Table 6:** Model identification and model estimation for the post hoc modified measurement model in Fig. 3 (applying robust DWLS and ML using the statistical package LISREL)

Model Identification		Unstandardized solution				Completely standardized solution	
		DWLS		ML		DWLS	ML
FP	Observed variables	Estimate	(SE)	Estimate	(SE)	Estimate	Estimate
1	CNL1 factor loading	.981	(.024)	.976	(.024)	.856	.852
	CNL2 factor loading	1.000*		1.000*		.872	.873
2	CNL3 factor loading	.932	(.020)	.930	(.020)	.813	.812
3	CNL4 factor loading	.932	(.023)	.933	(.023)	.813	.814
4	CNL5 factor loading	.931	(.021)	.931	(.021)	.812	.813
5	CNL1 unique variance	.267		.274		.267	.274
6	CNL2 unique variance	.239		.238		.239	.238
7	CNL3 unique variance	.339		.341		.339	.341
8	CNL4 unique variance	.339		.337		.339	.337
9	CNL5 unique variance	.340		.339		.340	.339
10	CNL1,CNL4 uniqueness relationship**	− 0.061	(.021)	− 0.059	(.021)	−.061	−.059
11	CNL2,CNL5 uniqueness relationship**	− 0.066	(.018)	−0.067	(.018)	−.066	−.067
	Latent variable						
12	CNL-Eval variance***	.761	.024	.762	.024	1.000	1.000

*Note*. CNL1 - CNL5 are the observed variables, CNL-Eval is the latent variable. FP = Free parameter (counting the number of free parameters to be estimated with reference to the unstandardized soultion), DWLS = Diagonally Weighted Least Squares estimation, ML = Maximum Likelihood estimation, SE = Standard Error, Factor loading = the proportion of the total variance that an item shares with the other items ie., is common to the items (a variance component accounted for by the latent variable in the model), Unique variance = the proportion of the total variance that is unique to an item (a variance component not accounted for by the latent variable model in the model i.e., the *un*modelled variance component). Additional correlation was specified between the error covariances of CNL1 and CNL4 and CNL2 and CNL5

#) Lisrel reports unique variance components as 1-*R*^2^ for both the standardized and the unstandardized solutions, where *R*^2^ is the squared standardized factor loading when the item only load on one factor

*) Factor loading constrained to 1 owing to item being used as reference or marker variable to resolve the origin and unit of measurement problem

**) The relationship refers to the covariance (in the *un*standardized solution) and the correlation (in the standardized solution) between the uniquene variance components of the repective observed variables. These relationships are data-driven re-specifications of M1

***) The variance of the latent variable is the “covariance with itself” in the *un*standardized solution and the “correlation with itself” in the standardized solution. The latter is always 1

## Discussion

Empirical data from the Rasch-modelling approach supports hypotheses H1 and H2 with one exception; the less complex parameterization of the polytomous unidimensional Rasch model (PCM) described the CNL-E scale data ‘significantly’ better than the more restricted rating scale parameterization (RSM) did. This means that the PCM contained more information about the data as it estimated one set of threshold parameters for each item, unlike the RSM, which estimates one set of step difficulties common for all items. We therefore offer the following post hoc explanation; that the four thresholds are not equal in size across the five items and there is need to estimate one set of threshold parameters for each item. And while using the same sample to evaluate fit of post hoc model modifications is not advised, we were not able to obtain another sample on which to test the modified model (M2). However we further justify these modifications based on the high negative residual correlations observed in Rasch analysis, indicative of items from different dimensions in the latent structure of the variable CNL-E.

Furthermore, qualitative interpretation of item content and categorization of the items into the subsets identified based on PCA residuals confirmed the substantive theory of the underlying latent trait (CNL-E); that critical evaluation of nutrition information from various sources requires skills that are well recognized and central to ‘media literacy’ and ‘scientific literacy’ [24, 61]. ‘Media literacy’ is concerned with skills pertaining to the ability to assess the consistency (reliability) of information while ‘scientific literacy’ is concerned with the skills that enable individuals to assess the the trustworthiness of information (validity of information) [21].

Higher item order on the latent continuum suggests that items addressing skills related to assessing the validity of information appear to provide more information about the latent trait (CNL-E) than those concerned with assessing the reliability of information. This supposition finds support in Potter’s cognitive model of media literacy [23], in which he describes the advancement in skills associated with the different levels of information-processing, starting with *filtering* of messages, analogous to assessing the reliability of information and sources; through to *meaning-matching*, *meaning-making* and finally *meaning-construction*. The latter steps, which point to advanced information-processing, require individuals to refer to previously learned knowledge in order to determine the meaning of a message and thereby create their own meaning that is relevant for them. Similarly, it can be anticipated that the ability to assess validity of nutrition information, requiring the ability to effectively interprete and use scientific knowledge as a criterion to appraise nutrition information from various sources like the media; reflects an advanced level of CNL evaluation.

### Limitations of the study

While it is recommended to evaluate fit of post hoc model modifications on a different sample in order to minimize the chances of capitalizing on sampling error, we were unable to obtain another sample on which to test the post hoc modified model. Therefore we recommend that the CNL-E scale is applied on different age-groups and populations in order give better insight into the validity of the modified model.

In the current study, the number of books owned at home was used as an indicator of family SES; while it is an appropriate indicator in studies involving young children and adolescents, it is rather outdated. With the widespread use of digital learning platforms including e-books, a better suited indicator of family SES could be the number of computers or e-readers that they have access to at home.

The sources of nutrition information that were captured by the items were limited to ‘traditional’ media and online media sources. Other information sources such as dietitians, peers, family could have been included, as it is equally important to establish the credibility of this information. Furthermore, rewording the items to remove complex jargon terms such as ‘claims’ might benefit the respondents who might not be familiar with the term.

## Conclusion

A significant *theoretical* outcome of our study is that we managed to overcome a well-known challenge of nutrition literacy measurement; the lack of a clear theoretical basis and thereby poorly founded methodological advancement [61].

An important practical outcome of this study was that we were able to develop a set of short non-abstract user-friendly test items assessing how individuals with a basic level of nutrition education (12th grade) evaluate nutrition information obtained from various sources. This is of significance as existing measures of critical evaluation of nutrition information are comprised of items which require the subjects to have an advanced knowledge about evidence-based medicine.

Furthermore, while it is recognized that measuring critical health literacy is demanding, requiring careful consideration of wording and context [61, 62]; the current study shows that by focusing on established aspects of the critical dimension of nutrition literacy such as ‘evaluation of nutrition information and advice’, it is possible to operationalize and measure nutrition literact at the ‘more advanced’ level (critical domain). Additionally, this study reveals the potential benefits of critical thinking skills in effective evaluation of nutrition information from various sources. By emphasizing the skillful analyzing, translating and application of established scientific knowledge across different disciplines like nutrition; individuals will be better equipped to identify potentially harmful nutrition claims, thereby lessening the ‘confusion’ caused by the seemingly contradicting nutrition information from various sources.

Lastly, as the field of nutrition literacy advances, applying instruments such as the valid, accurate and precise CNL-E scale presented in this paper, that may be beneficial in evaluating the impact of interventions and programs that are primarily focused on nutrition education.

## Abbreviations

AIC: Akaike Information criteria
CFA: Confirmatory factor analysis
CFI: Comparative fit index
CNL: Critical nutrition literacy
CNL-E: Critical Nutrition Literacy-Evaluation
*df*: Degrees of freedom
DIF: Differential item functioning
DWLS: Diagonally weighted least squares
FNL: Functional nutrition literacy
ICC: Item characteristic curve
INL: Interactive nutrition literacy
LRT: Likelihood ratio test
ML: Maximum likelihood
NNFI: Non-normed fit index
PCM: Partial credit parameterization of the Rasch model
PCMRSM: Rating scale parametrization of the Rasch model
PSI: Person Separation Index
RMSEA: Root mean square error of approximation
SB: Satorra-Bentler
SRMR: Standardized root mean square residual

## Footnotes

1 No item unique correlations were specified a priori based on prior research in different samples. The FP increases to 12 and *df* decreases to 3 after specifying 2 unique correlations post hoc (model in Fig. 3).

## References

[1] Yang CC, Chen H, Honga K (2003). Visualization of large category map for internet browsing. Decis Support Syst.

[2] Shirky C. It's not information overload. It's filter failure. Mas Context. 2014. http://mascontext.com/pdf/MAS_Context_Issue07_INFORMATION.pdf. Accessed 16 July 2017.

[3] Ishikawa H, Kiuchi T. Health literacy and health communication. Biopsychosoc Med. 2010. 10.1186/1751-0759-4-18. 10.1186/1751-0759-4-18 PMC2990724 21054840

[4] Sørensen K, Van den Broucke S, Fullam J, Doyle G, Pelikan J, Slonska Z, Brand H. Health literacy and public health: a systematic review and integration of definitions and models. BMC Public Health. 2012. 10.1186/1471-2458-12-80. 10.1186/1471-2458-12-80 PMC3292515 22276600

[5] Neuhauser L, Rothschild R, Rodríguez FM (2007). MyPyramid.gov: assessment of literacy, cultural and linguistic factors in the USDA food pyramid web site. J Nutr Educ Behav.

[6] Silk KJ, Sherry J, Winn B, Keesecker N, Horodynski MA, Sayir A (2008). Increasing nutrition literacy: testing the effectiveness of print, web site, and game modalities. J Nutr Educ Behav.

[7] Zoellner J, Connell C, Bounds W, Crook L, Yadrick K (2009). Nutrition literacy status and preferred nutrition communication channels among adults in the lower Mississippi Delta. Prev Chronic Dis.

[8] Guttersrud Ø, Pettersen KS. Young adolescents’ engagement in dietary behaviour–the impact of gender, socio-economic status, self-efficacy and scientific literacy. Methodological aspects of constructing measures in nutrition literacy research using the Rasch model. Public Health Nutr. 2015. 10.1017/S1368980014003152. 10.1017/S1368980014003152 PMC10271695 25634262

[9] Diamond JJ. Development of a reliable and construct valid measure of nutritional literacy in adults. Nutrition. 2007. 10.1186/1475-2891-6-5. 10.1186/1475-2891-6-5 PMC1804274 17300716

[10] Krause C, Sommerhalder K, Beer-Borst S, Abel T. Just a subtle difference? Findings from a systematic review on definitions of nutrition literacy and food literacy. Health Promot Int. 2016:daw084. 10.1093/heapro/daw084 PMC6005107 27803197

[11] Rothman RL, Housam R, Weiss H, Davis D, Gregory R, Gebretsadik T, Elasy TA (2006). Patient understanding of food labels: the role of literacy and numeracy. Am J Prev Med.

[12] TenHave TR, Van Horn B, Kumanyika S, Askov E, Matthews Y, Adams-Campbell LL (1997). Literacy assessment in a cardiovascular nutrition education setting. Patient Educ Couns.

[13] Weiss BD, Mays MZ, Martz W, Castro KM, DeWalt DA, Pignone MP, Hale FA (2005). Quick assessment of literacy in primary care: the newest vital sign. Ann Fam Med.

[14] Carbone ET, Zoellner JM (2012). Nutrition and health literacy: a systematic review to inform nutrition research and practice. J Acad Nutr.

[15] Velardo S (2015). The nuances of health literacy, nutrition literacy, and food literacy. J Nutr Educ Behav.

[16] Guttersrud Ø, Dalane JØ, Pettersen S (2014). Improving measurement in nutrition literacy research using Rasch modelling: examining construct validity of stage-specific ‘critical nutrition literacy’ scales. Public Health Nutr.

[17] Nutbeam D (2000). Health literacy as a public health goal: a challenge for contemporary health education and communication strategies into the 21^st^ century. Health Promot Int.

[18] Berman M, Lavisso-Mourey R (2008). Obesity prevention in the information age. J Am Med Assoc.

[19] Schulz P, Nakamoto K (2005). Emerging themes in health literacy. Stud Commun Sci.

[20] Organisation for Economic Co-operation and Development (OECD). Assessing scientific, reading and mathematical literacy: A framework for PISA 2006. OECD, Paris: 2006.

[21] Pettersen K.S. Health claims and scientific knowledge. A study of how students of Health Sciences, their teachers and newspaper journalists relate to health claims in society. University of Oslo, Oslo: 2007.

[22] Aufderheide P, Firestone C. Media Literacy: A report of the national leadership conference on media Literacy Queenstown MD: Aspen Institute; 1993.

[23] Potter WJ. Theory of media literacy: A cognitive approach. London: Sage Publications Inc; 2004.

[24] Frisch AL, Camerini L, Diviani N, Schulz PJ (2012). Defining and measuring health literacy: how can we profit from other literacy domains?. Health Promot Int.

[25] Paakkari L, Paakkari O (2012). Health literacy as a learning outcome in schools. Health Educ.

[26] Thomson S, Hillman K, De Bortoli L. A teacher’s guide to PISA scientific literacy. ACER press, Australia: 2013.

[27] Ministry of Education and Research, Norway: The Education System. https://www.regjeringen.no/en/topics/education/school/the-norwegian-education-system/id445118/. Accessed 17 Apr 2018.

[28] Aikens NL, Barbarin O. Socioeconomic differences in reading trajectories: the contribution of family, neighborhood, and school contexts. J Educ Psychol. 2008;100. 10.1037/0022-0663.100.2.235.

[29] Foster MA, Lambert R, Abbott-Shim M, McCarty F, Franze S (2005). A model of home learning environment and social risk factors in relation to children’s emergent literacy and social outcomes. Early Child Res Q.

[30] Bergen E, Zuijen T, Bishop D, Jong PF (2017). Why are home literacy environment and children’s reading skills associated? What parental skills reveal. Read Res Q.

[31] Skolverket. Nationella utvärderingen av grundskolan 2003 [The National evaluation of the compulsory school]. Stockholm: Skolverket; 2004.

[32] Marais I, Andrich D (2008). Formalizing dimension and response violations of local independence in the unidimensional Rasch model. J Appl Mes.

[33] Tennant A, Conaghan PG (2007). The Rasch measurement model in rheumatology: what is it and why use it? When should it be applied and what should one look for in a Rasch paper?. Arthritis Care Res.

[34] Adams R, Wu M. Multidimensional models.In ConQuest tutorial. 2010. Retrieved from https://www.acer.org/files/Conquest-Tutorial-7-MultidimensionalModels.pdf.

[35] Rasch G, Lazarsfeld PF, Henry NW (1966). An individualistic approach to item analysis. Readings in mathematical social science. Chicago: science research associates.

[36] Andersen EB (1977). Sufficient statistics and latent trait models. Psychometrika.

[37] Salzberger T (2010). Does the Rasch model convert an ordinal scale into an interval scale. RMT.

[38] Andrich D. Rasch models for measurement 1988. Beverly Hills: Sage.

[39] Perline R, Wright BD, Wainer H (1979). The Rasch model as additive conjoint measurement. Appl Psych Meas.

[40] Rasch G. Probabilistic models for some intelligence and attainment tests. 1983 ed. Copenhagen: Danish institute for Educ Res; 1960.

[41] Dunn OJ. Estimation of the medians for dependent variables. Ann Math Stat. 1959. 10.1214/aoms/1177706.

[42] Walker CM, Beretvas SN, Ackerman T (2001). An examination of conditioning variables used in computer adaptive testing for DIF analyses. Appl Meas Educ.

[43] Cronbach LJ. Coefficient alpha and the internal structure of tests. Psychometrika. 1951; 10.1007/BF02310555.

[44] RUMM Laboratory Pty Ltd, Interpreting RUMM2030: part III estimation and statistical techniques, 2009.

[45] Andrich D (1982). An index of person separation in latent trait theory, the traditional KR. 20 index, and the Guttman scale response pattern. ERP.

[46] RUMM Laboratory Pty Ltd, Differential item function (DIF) analyses in extending the RUMM2030 analysis, November 2009.

[47] RUMM Laboratory Pty Ltd, Quantifying response dependence in extending the RUMM2030 analyses, November, 2009.

[48] Wright B (1996). Local dependency, correlations and principal components. RMT..

[49] Hagell P (2014). Testing rating scale Unidimensionality using the principal component analysis (PCA)/t-test protocol with the Rasch model: the primacy of theory over statistics. OJS.

[50] Smith EV (2002). Understanding Rasch measurement: detecting and evaluating the impact of multidimensionality using item fit statistics and principal component analysis of residuals. JAM.

[51] ACER ConQuest command reference, July 2015.

[52] Joreskog K, Sorbom D. LISREL (Version 9.3). 2009; Chicago: Scientific Software: Inc.

[53] Teo T, Tsai LT, Yang CC. Applying structural equation modeling (SEM) in educational research. In: Myint SK, editor. Application of structural equation modelling in educational research and practice. Sense: Publishers; 2013. p. 3–21.

[54] Diamantopoulos A, Siguaw JA. Introducing LISREL. A guide for the uninitiated. London: SAGE publications; 2000.

[55] Bandalos DL, Sara JF. Factor analysis: Exploratory and confirmatory. In: Mueller RO, Hancock GR, editors. The Reviewer's guide to quantitative methods in the social sciences. Routledge; 2010.

[56] Browne MW, Cudek R. Alternative ways of assessing model fit. In: Bollenand KA, Long JS, editors. Testing structural equation models. Newbury Park: SAGE; 1993.

[57] Hoelter JW (1983). The analysis of covariance structure: goodness of fit indices. Sociological methods and Research.

[58] MacCallum R.C. 1995. Model specification: procedures, strategies and related issues. In: Hole RH, editor. Struct Equ Model: Concepts, issues and applications pp. 16–36. Thousand Oaks: Sage; 1995.

[59] Martens MP (2005). The use of structural equation modelling in counselling psychology research. Couns Psychol.

[60] MacCallum RC, Roznowski M, Necowitz LB (1992). Model modifications in covariance structure analysis: the problem of capitalization on chance. Psychol Bull.

[61] Steckelberg A, Hülfenhaus C, Jürgen K, Jürgen R (2009). Mühlhauser. How to measure critical health competences: development and validation of the critical health competence test (CHC test). Adv in health. Sci Educ.

[62] Krause C, Sommerhalder K, Beer-Borst S (2016). Nutrition-specific health literacy: development and testing of a multi-dimensional questionnaire. Ernahrungs-Umschau.

